# Individual differences in fear memory expression engage distinct functional brain networks

**DOI:** 10.1101/2025.05.12.653531

**Published:** 2025-07-28

**Authors:** Barbara D. Fontana, Jacob Hudock, Neha Rajput, Dea Kanini, Dinh Luong, Justin W. Kenney

**Affiliations:** Department of Biological Sciences, Wayne State University, Detroit, MI 48202

## Abstract

Fearful stimuli elicit a mix of active (*e.g.,* evasion) and passive (e.g., freezing) behaviors in a wide range of species, including zebrafish (*Danio rerio*). However, it is not clear if individual differences in fear responses exist and, if so, what parts of the brain may underlie such differences. To probe these questions, we developed a contextual fear conditioning paradigm for zebrafish that uses conspecific alarm substance (CAS) as an unconditioned stimulus where fish associate CAS administration with a specific tank. To identify individual differences, we collected behavioral responses from over 300 fish from four different strains (AB, TU, TL, and WIK) and both sexes. We found that fear memory behavior fell into four distinct groups: non-reactive, evaders, evading freezers, and freezers. We also found that background strain and sex influenced how fish respond to CAS, with males more likely to increase evasive behaviors than females and the TU strain more likely to be non-reactive. Finally, we performed whole-brain activity mapping to identify the brain regions that are associated with different behavioral responses. All groups exposed to the tank had strong engagement of the telencephalon, whereas regions beyond the telencephalon distinguished behavioral groups: animals that have high levels of freezing, but low levels of evasion, uniquely engage the cerebellum, preglomerular nuclei, and pretectal areas, whereas those fish that mix evasion with freezing engage the preoptic and hypothalamic areas. Taken together, these findings reveal that zebrafish exhibit individual differences in fear memory expression that are supported at the neural level by extra-telencephalic regions.

## Introduction

Fear is an emotional state elicited by perceived threat. Behaviorally, fear manifests as stereotypical responses that have a striking similarity across a range of taxa (Adolphs, 2013; Darwin, 1872). These responses can be classified into two broad categories: active (e.g., fighting or fleeing) and passive (e.g., freezing or immobility). The choice of the appropriate behavior in a given circumstance is critical to survival. For example, evasive behaviors may be ideal when a predator is actively striking whereas freezing (i.e., a lack of movement) may be best when a predator is thought to be lurking. Recalling where fearful stimuli have been experienced is key to avoiding predation and maintaining survival, enabling animals to anticipate danger and optimize survival strategies (Mobbs et al., 2015). While fear conditioning is well studied across species, the extent to which individuals differ in the expression of fear responses, and how these behavioral differences are represented in the brain, remains poorly understood.

One of the most reliable and ethologically relevant elicitors of fear in animals are alarm pheromones. Pheromones are small molecules that are released by animals as a form of communication and can be used to signal the presence of a threat (Tirindelli et al., 2009). The existence of alarm pheromones is well established in many species of insects, fish, and mammals (Verheggen et al., 2010) and is increasingly recognized as existing in humans (de Groot and Smeets, 2017). In fish, the first description of alarm pheromone was by von Frisch (von Frisch, 1938) in minnows (*Phoxinus laevis*), where he described how an extract of epithelial cells resulted in fear-related behaviors like freezing and erratic swimming. Alarm pheromone is also species specific: fish preferentially respond to conspecific alarm substance (CAS) while ignoring alarm substances from other species. He also found that fish formed memories of where they previously experienced exposure to CAS and that individuals sometimes varied in their behavioral responses. More recently, variability in responsiveness to CAS has been reported in a range of fish species (Pfeiffer, 1977) including zebrafish (*Danio rerio*) (Speedie and Gerlai, 2008; Suboski et al., 1990). These findings are suggestive of the presence of consistent individual behavioral differences in fear reactivity, but such differences have not been systematically examined.

Given the ethological relevance of CAS as a stimulus, it holds considerable potential for use in understanding individual variation in associative fear learning (i.e., Pavlovian fear conditioning) in zebrafish. In fear conditioning, a conditioned stimulus (CS) is paired with an intrinsically aversive unconditioned stimulus (US). Later presentation of the CS results in a conditioned response that is a measure of the strength of the memory. The CS can be either a discrete cue, like a light or sound, or a diffuse stimulus, like a tank or context. CAS has had success as a US in Pavlovian fear learning assays in zebrafish where it has been associated with lights, odors, and contexts (Baker and Wong, 2019; Hall and Suboski, 1995; Maximino et al., 2018a; Ruhl et al., 2017). These data suggest that fear learning using CAS is a robust phenomenon in zebrafish, making it ideal for determining if individual animals vary in fear memory expression and identifying the corresponding neural substrates.

To identify the presence of individual differences in fear behavior, we collected data from over 300 adult zebrafish from four inbred strains and both sexes. To increase throughput, we automated behavioral data analysis by combining DeepLabCut (Mathis et al., 2018) with a random forest supervised machine learning model (James et al., 2013). We then used unsupervised machine learning to identify the presence of individual differences in fear memory associated behaviors. Lastly, we performed whole-brain activity mapping during fear memory recall. We found that brain regions outside the telencephalon, such as the cerebellum, preglomerular nuclei, and hypothalamus, are most strongly associated with different fear memory behavioral phenotypes.

## Results

### Automated behavioral identification

**Figure 1.**
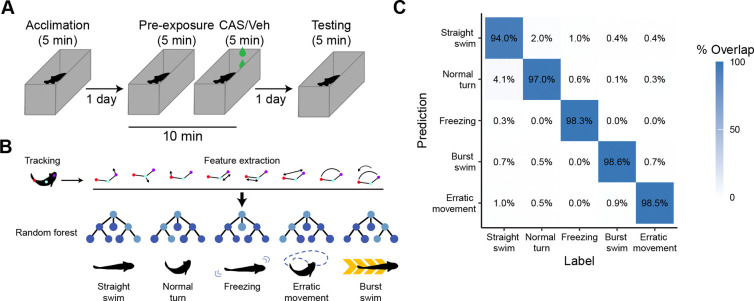
Fear memory paradigm and automated behavioral assessment. A) Adult zebrafish were trained in contextual fear conditioning over three days using CAS as the unconditioned stimulus. B) Automated behavioral assessment began with tracking using DeepLabCut, followed by feature extraction and then training of a random forest model for classification. C) Confusion matrix for the random forest model using a model trained on 80% of the data and tested on 20% of the data not used for training.

To assess contextual fear memory in zebrafish we designed a three-day behavioral paradigm (Figure 1A). On the first day, animals were given five minutes to acclimate to the tank. On the second day, animals were placed in the tank for 10 minutes. Baseline behavior was captured during the first five minutes (i.e., pre-exposure). CAS or vehicle was then added to the tank for five additional minutes. On the third day, fish were placed back in the same tank in the absence of CAS to assess fear memory. To automate the identification of different swimming behaviors, we trained a DeepLabCut (Mathis et al., 2018) network to track three points on the fish: the head, trunk, and tail (Figure 1B). We then used the output from this tracking to train a random forest model to identify five different behaviors: straight swimming, normal turns, freezing, burst swimming, and erratic movements. We used 34 parameters derived from the position tracking to capture the movement of the animals over time (Table S1). These features were calculated using a sliding window of 45 frames (750 ms) to capture the temporal dynamics of movement. Approximately 14,000 frames were labelled for each of five different behaviors, with a 80/20 split for training/testing. We optimized the number of trees and number of features per tree, finding that 2,000 trees and 10 variables per tree optimized the out of bag error rate (2.88%). The resulting model had at least 94% accuracy (Figure 1C). Notably, most of the misclassifications were between straight swimming/normal turns and burst swimming/erratic movements. Because these labels capture the same behavioral concept, and to simplify the subsequent analysis, we typically use ‘normal swimming’ to refer to either straight swimming or normal turns and ‘evasive behavior’ to capture erratic movement or burst swimming.

### Contextual fear learning

**Figure 2.**
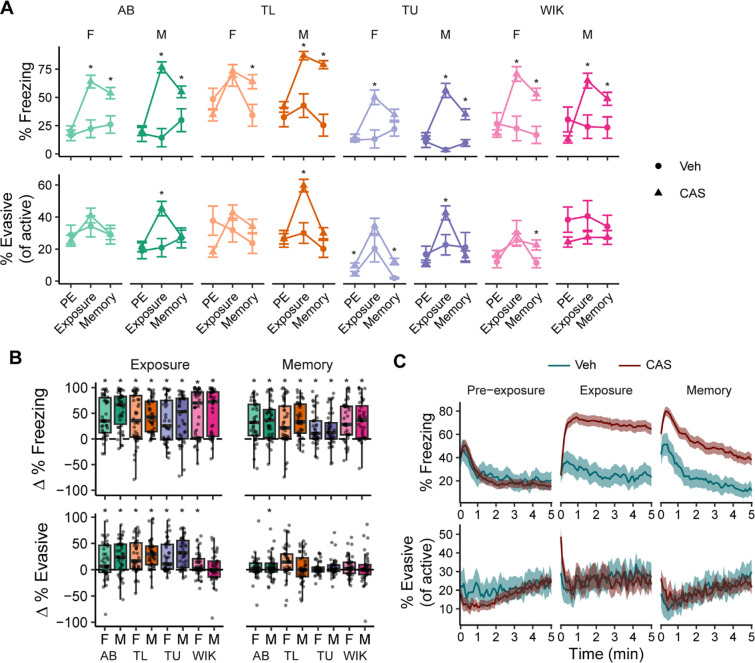
Behavioral responses during fear conditioning. A) Percent freezing (above) and % evasive behavior as a proportion of active behavior (below). Data are means ± SEMs. *-P < 0.05 compared to vehicle treated fish via t-tests. B) Change (in comparison to pre-exposure) in percent freezing (above) and percent evasive behavior (below) during exposure to CAS (left) and during memory day (right). *-P < 0.05 compared to zero via one-sample t-tests. Boxplot center is the median, hinges are interquartile range, and whiskers are the hinge ± 1.5 times the interquartile range. C) Freezing (above) and evasive behavior as proportion of active (below) behavior over time during pre-exposure, exposure, and memory days. Lines are means and ribbons are 95% confidence intervals. Vehicle treated groups, n’s = 12; CAS treated groups, n’s = 40–42. Part C includes all the CAS treated animals (N = 331).

Given prior reports of behavioral variation in response to CAS, we hypothesized that this variation may be due to background strain and/or sex of the animals. When looking at how fish respond to the CAS itself, we found that almost all strains and sexes had an increase in freezing compared to vehicle treated animals (Figure 2A, top; detailed statistics in **File S1**), and all groups were elevated relative to pre-exposure (Figure 2B, top; detailed statistics in **File S2**). The only exception to this was female TL fish where the vehicle treated animals also increased their freezing behavior, likely as a response to the small disturbance that occurs due to the addition of a solution to the water. Evasive behaviors in response to CAS had more variation. Compared to vehicle treated animals, CAS treated males in three out of four strains (except WIKs) increased evasive behavior (Figure 2A, bottom) whereas there were no changes in females. Compared to pre-exposure, CAS resulted in an increase in evasive behaviors in all groups except male WIKs (Figure 2B, bottom).

We next asked whether fish formed a long-term associative memory between the tank and CAS. To test this, fish were placed back into the exposure tank one day later in the absence of CAS. On memory day, there was elevated freezing compared to vehicle treated animals and compared to pre-exposure in nearly all strains and sexes, with the exception of female TU fish that did not differ from their vehicle treated counterparts (Figures 2A and 2B, top). As before, evasive behaviors were much more variable on memory day. When compared to vehicle treated animals, evasive behavior was elevated in only the females of two strains, TUs and WIKs (Figure 2A, bottom); compared to pre-exposure, only male ABs had an increase in evasive behaviors (Figure 2B, bottom).

To better understand the dynamics of freezing and evasive behavior, we examined their temporal profiles during the different stages of the paradigm (Figure 2C). During pre-exposure, there was an initial increase in freezing behavior that quickly decreased to ~20%; evasive behavior remained consistently low throughout the trial. During exposure, CAS treated animals had an initial burst in evasive behavior that rapidly gave way to persistent freezing. On memory day, we saw an initial increase in freezing behavior in both vehicle and CAS treated animals, but while the freezing in vehicle treated animals quickly declined, the freezing in CAS treated animals was higher and persisted for longer (Figure 2C). Evasive behavior during memory was largely similar between vehicle and CAS treated animals, with only a slight increase in CAS treated animals at the beginning of the trial.

### Transitions between behavioral states

**Figure 3.**
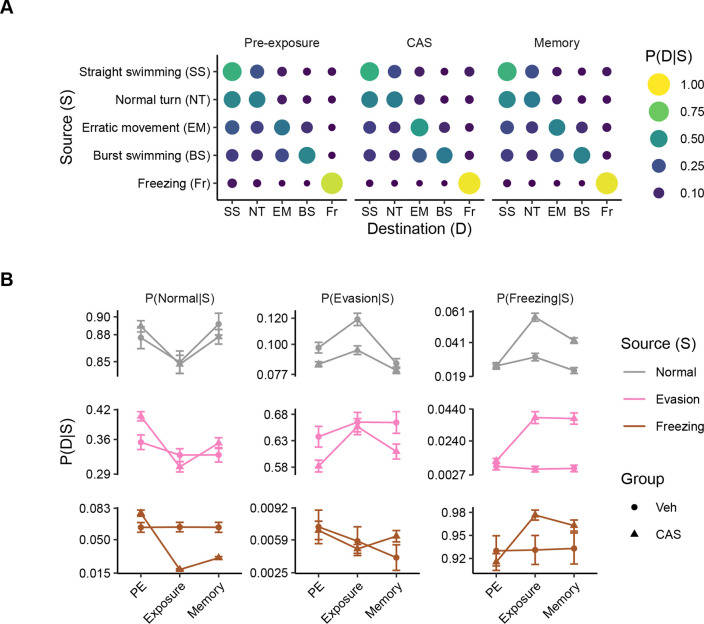
Transitions between behavioral states. A) Transition matrices capturing how often zebrafish change from the source to the destination state at different stages of the contextual fear memory behavioral paradigm. B) Transitions between source and destination states over time for normal, evasive and freezing behavior. Error bars are 95% confidence intervals.

The aggregate data we have examined so far does not yield insight into how fish switch between behavioral states. To examine these state changes, we constructed transition matrices (Figure 3). We found that at all stages, freezing is the most stable behavior (i.e., the behavior is most likely to follow itself), even during pre-exposure when it is a relatively small proportion of the overall behavior (Figure 3A). Amongst the normal swimming behaviors of straight swimming and normal turns, transitions are most likely between these states across all stages of behavior. A similar pattern is seen for evasive swimming (i.e., erratic movements and burst swimming), however, these behaviors are less likely to occur after normal swimming than normal swimming is to occur after evasive behavior. Overall, there are no large changes in the patterns of transitions across the different stages of the behavioral paradigm. These data suggest that exposure to CAS does not result in wholesale reorganization of behavior, but instead a change in how much of each behavior is selected.

To capture more subtle behavioral changes, we examined state transitions in greater detail while collapsing straight and normal turns into ‘normal swimming’ and burst swimming and erratic movements into ‘evasive behavior’ for clarity (Figure 3B). Here, we see that during CAS treatment, there is a small destabilization of normal behavior such that normal behavior becomes less likely following itself and is more likely to followed by freezing (Figure 3B, top row). In contrast, CAS causes freezing behavior to become much more stable, as it is more likely to occur following normal, evasive, and freezing behavior at both exposure and memory day (Figure 3B, right column). Evasive behavior only increases in likelihood following itself during CAS treatment, with little to no change following normal or freezing behavior (Figure 3B, middle column). Taken together, these data suggest that the increase in evasive behavior during CAS exposure is due to an increase in the length of evasive behavior bouts, but not to an increased likelihood of transition to evasion from other behavioral states. In contrast, the elevation in freezing behavior during exposure and memory is due to both an increase in both the duration and number of freezing bouts.

### Individual differences in fear memory expression

**Figure 4.**
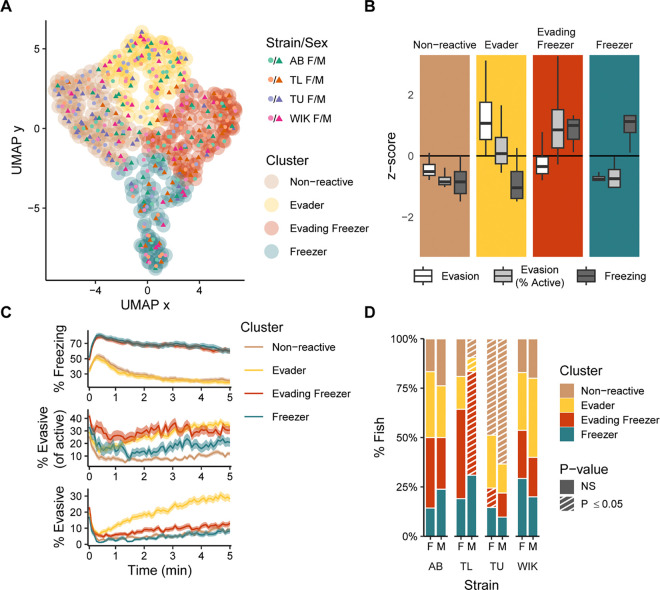
Clustering of zebrafish behavior during fear memory recall. A) Two-dimensional representation of the three-dimensional behavioral space using a uniform manifold approximation (UMAP). The outer circles delineate the clusters as defined using Louvain clustering applied to a k-nearest neighbor network (k = 74). Each point represents an individual fish (N=331). B) Behaviors associated with the identified clusters presented as z-scores. Box plots indicate median (center line), interquartile range (box ends) and ± 1.5 times the interquartile range (whiskers). C) Behaviors across time for the different clusters; ribbons indicate 95% confidence intervals. D) Percentage of animals falling into each behavioral cluster across strain and sex. Striped bars (P < 0.05) represent under/over representation using permutation tests with FDR corrections; n’s = 40–42 per strain and sex.

Given the large amount of variation in behavioral responses to CAS, especially during memory day (Figure 2B), we hypothesized the presence of distinct fear memory behavioral types. To uncover these types, we applied Louvain clustering to a series of k-nearest neighbor networks and identified the k-value that yielded the most robust and best clustering (k = 74; Figure S2) (Rajput et al., 2022). Distances between nodes (i.e., individual fish) in the network were calculated using percent evasion, percent evasion of active swimming, and percent freezing. This resulted in four behavioral clusters (Figure 4A). We named these clusters (Figure 4B): (1) “non-reactive”: fish low in evasion and freezing; (2) “evaders”: fish low in freezing and high in overall levels of evasive behavior; (3) “evading freezers”: fish high in freezing and evasive behaviors; (4) ‘freezers’: fish high in freezing and low in evasive behaviors.

Temporal profiles of behavior also reveal distinct differences (Figure 4C). For example, both freezer groups overlapped in the percent freezing behavior over time but clearly differ in their evasive behavior, whereas the evading freezers have both higher overall levels of evasion and a greater proportion of evasion relative to active behavior throughout the entire memory trial. Likewise, the temporal profile of freezing for the evaders and non-reactive fish are equally low, but the evaders slowly increase their evasive behaviors over time whereas non-reactive fish remain low in evasion throughout the trial.

Finally, we asked whether behavioral types are affected by the strain and/or sex of the fish (Figure 4D). We found that in AB and WIK fish there was neither over nor underrepresentation in any of the four behavioral types. In contrast, TU fish were overrepresented in the non-reactive group, and TL males were overrepresented in the evading freezer group.

### Behavioral clusters over time

**Figure 5.**
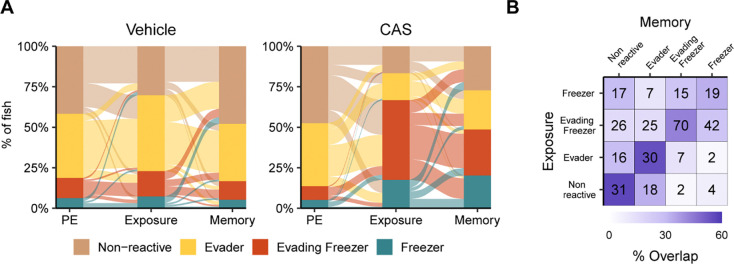
Transitions between behavioral states over time. A) Alluvial plot indicating the number of fish in each cluster during pre-exposure, exposure, and memory day for animals exposed to vehicle (left) or CAS (right). B) Confusion matrix of animals falling into different clusters during exposure and memory day. Numbers indicate the quantity of animals falling into each group on exposure and memory days. Vehicle treated fish; N = 96; CAS treated: N = 331.

We next asked if behavior during exposure to CAS is predictive of behavior at memory day. To do this, we identified which cluster each fish fell into at each behavioral stage (Figure 5A). In the vehicle treated group, animals were largely identified as either non-reactive or evaders at all three time points. In CAS treated fish, only 14% of animals belonged to one of the freezing groups at pre-exposure. During CAS exposure, this proportion increased to 66%. On memory day, 48% of CAS treated fish were in one of the two freezing groups, about equally split between evading freezers (28%) and freezers (20%). We also found that the behavioral cluster of the fish on exposure day roughly predicted behavior on memory day (Figure 5B, diagonal). Most fish in the non-reactive and evader groups on memory day fell into the same category during exposure (54–56%) or transitioned between evaders and the non-reactive groups (29%). Evaders and non-reactive fish rarely transitioned to freezing groups on memory day (11–16%). In contrast, a substantial minority of animals in the freezing groups transitioned to non-freezing groups (30–41%) with the majority remaining in one of the high freezing clusters (58–69%). Taken together, these data suggest that fish behavior is largely consistent between exposure and memory days, although there remains a sizable minority of fish that switch from freezing to non-freezing behaviors at memory day.

### Brain-behavior covariation

**Figure 6.**
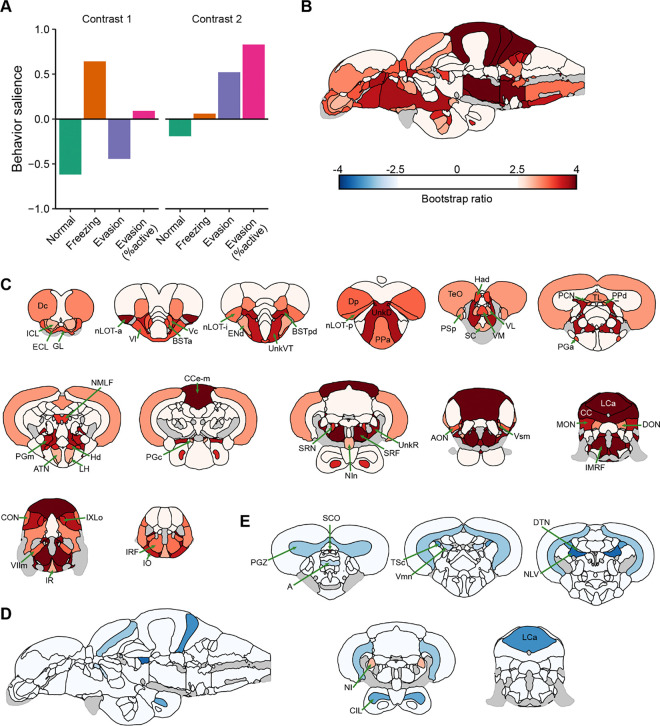
Partial least squares (PLS) analysis to identify brain regions that covary with behavior. A) Top two contrasts and related behavioral saliences identified from PLS analysis. B) Bootstrap ratios of brain saliences for the first contrast on a sagittal image of the adult zebrafish brain. Only bootstrap ratios of above 2.5 are depicted. C) Coronal slices with same coloration as part B. D) Bootstrap ratios of brain saliences for the second contrast on a sagittal image. E) Coronal slices of bootstrap ratios correspond to the second contrast. Brain region abbreviations can be found in Table S2. N = 87.

To determine the brain regions that covary with behavior, we performed whole-brain activity mapping followed by partial least squares (PLS) analysis. We chose the AB strain of fish because they showed approximately equal distribution of the different behavioral types (Figure 4D). For brain mapping, animals were euthanized 15 minutes after fear memory recall and processed for tissue clearing using iDISCO. Neural activity was captured using *in situ* hybdridization chain reaction (HCR) against *cfos* mRNA. Brains were imaged using light-sheet microscopy and then registered to the adult zebrafish brain atlas (AZBA) (Kenney et al., 2021; Rajput et al., 2025). We used PLS analysis (Krishnan et al., 2011) to capture covariation between brain activity and four different behaviors: normal swimming, freezing, evasion, and evasion as percent of active behavior (Figure 6). The first behavioral contrast was significant (P = 0.0090) and captured freezing in the positive and evasion/normal swimming in the negative direction (Figure 6A, left); the second contrast approached, but did not reach significance (P = 0.107; Figure 6A, right). However, because this second contrast uniquely captured evasive behavior, we analyzed it with the caveat that these findings are not as strongly supported as those for the first contrast.

To determine which brain regions consistently contributed to the first behavioral contrast, we calculated bootstrap ratios (i.e., salience scores) that capture a combination of the magnitude and stability of regional contributions to a contrast (McIntosh and Lobaugh, 2004). For the first contrast (Figures 6B and C), we found several brain regions that significantly contributed to covariation (bootstrap ratios ≥ 2.5). The cerebellum stands out with regions such as the CC (cerebellar crest), CCe-m (molecular layer of the corpus cerebelli), MON (medial octavolateralis nucleus), and LCa (caudal lobe of the cerebellum) all with bootstrap ratios over 3.5 (Figure S3A and **File S6**). We also found several regions of the telencephalon that covaried with freezing, such as the nuclei of the lateral olfactory tract (nLOTs), parts of the bed nucleus of the stria terminalis (BSTs), the central and lateral portions of the ventral telencephalon (Vc and Vl), and the posterior and central portions of the dorsal telencephalon (Dp and Dc). The medial portion of the dorsal telencephalon (Dm), considered the zebrafish homolog to the mammalian basolateral amygdala (Porter and Mueller, 2020) also contributed to covariation, but to a lesser extent than other parts of the telencephalon (bootstrap ratio = 2.42).

For the second contrast (Figures 6D, 6E and S3B), there were only two regions that contributed to covariance with evasive behavior: the nucleus isthmi (NI) and the subcomissural organ (SC) (bootstrap ratios ≥ 2.5). Several regions negatively covaried with evasive behavior and thus are likely important for normal swimming, such as the parts of the medulla (Vmn: mesencephalic nucleus of the trigeminal nerve; and NLV: nucleus of the lateralis valvulae) and the tegmentum (NLL: nucleus of the lateral lemniscus and DTN: dorsal tegmental nucleus).

### Brain networks underling individual differences in behavior

**Figure 7.**
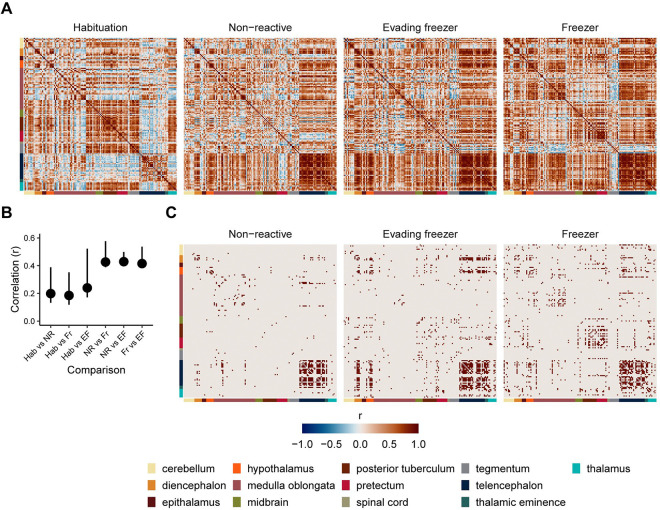
Covariation between brain regions across behavioral groups. A) Correlation matrices of cfos activity between brain regions. Each row/column is an individual brain region, and each entry is the Pearson correlation of cfos activity across animals in a group. Colored bars represent the ontological level of each brain region. B) Pearson’s correlations of matrices from each group with one another. Error bars are bootstrapped 95% confidence intervals. C) Correlation matrices with only suprathreshold correlations (FDR < 0.001). Habituation: n = 19; non-reactive, n = 17; evading freezer: n = 16; freezer: n = 21.

The brain is best thought of as a network of interacting regions whose complex interactions give rise to behavior. To determine the patterns of brain activity that are present during different types of fear memory expression, we generated networks from correlated *cfos* activity between brain regions for each experimental group (Figure 7). The overall pattern of correlations (Figures 7A & B) is similar in the groups exposed to the behavioral tank (i.e., non-reactive, evading freezer, and freezer groups; similarity of matrices: r’s=0.41–0.43) while clearly differing from the habituation group that was not exposed to the behavioral tank (r’s=0.18–0.24). To identify which regional interconnections were significant for network generation, we applied a threshold of FDR < 0.001 (Figure 7C). This identified fewer significant correlations in the non-reactive group (306) than the evading freezer (588) and freezer groups (655), suggesting these latter two groups form a more coherent brain network.

**Figure 8.**
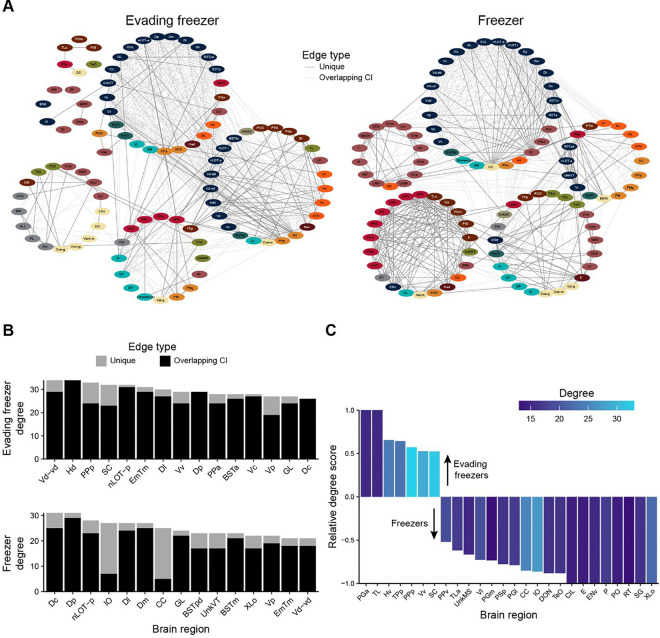
Functional brain networks underlying fear memory expression. A) Functional networks where nodes represent brain regions, and edges the presence of suprathreshold correlations (FDR < 0.001). Node colors correspond to ontological levels from figure 7. Lighter dashed edges correspond to suprathreshold correlations where confidence intervals overlap with the correlation from at least one other network (evading freezer, freezer, or non-reactive). Solid lines correspond to suprathreshold correlations with no overlaps with correlations in other matrices. B) Degree centrality of the top 15 nodes. Bars are colored according to the number of edges that arise from unique (light gray) or overlapping (black) confidence intervals as in part A. C) The relative degree score for nodes enriched in the evading freezer (positive) or freezer (negative) functional networks. Only those nodes with a relative degree score of more than ± 0.5 and degree greater than 12 are shown.

To generate insight into how the patterns of brain activity in the evading freezer and freezer groups differed, we generated functional networks (Figure 8). In these networks, the nodes represent brain regions and edges represent the presence of a suprathreshold correlation (Figure 7C). To better visualize how the networks in these two behavioral groups differed, we identified edges whose confidence intervals did not overlap with those of the other behavioral network or the non-reactive network (Figure 8A, solid lines). Significant edges/correlations that had overlapping confidence intervals are depicted as dashed lines. In examining the networks, we see that both networks have strong interconnectivity amongst regions of the pallium and subpallium, which is largely shared with the non-reactive group (Figure S4). In the evading freezer functional network, we see a greater engagement of several hypothalamic regions (orange) with several parts of the ventral telencephalon (blue). Whereas in the freezer functional network, the corpus cerebelli (CC) and inferior olive (IO) stand out as having many unique connections with the dorsal and ventral telencephalon. Freezers also have dense interconnectivity between the pretectum (red) and posterior tuberculum (light brown).

These qualitative observations of the network were confirmed by the degree centrality of each network (Figure 8B). In the evading freezer network, four regions in the ventral telencephalon (Vd-vd, Vv, Vc, and Vp) ranked in the top 15. In the hypothalamus, the dorsal hypothalamus (Hd) ranked highly in degree and the ventral hypothalamus (Hv) had greater degree in the evading freezer than the freezer network (Figure 8C). Several regions of the preoptic area also stand out in the evading freezer network, such as the anterior and posterior preoptic regions (PPa and PPp) and suprachiasmatic nucleus (SC), all of which are in the top 15 for degree centrality. Notably, the PPp and SC are also both high in relative degree (Figure 8C) and have more than 25% of their edges as unique to the evading freezer network (Figure 8B).

In the freezer functional network, the CC and IO are amongst those with the highest overall and relative degree and both have over 74% of their edges as being unique to the freezer network (Figures 8B and C). Several regions of both the pretectum (PO, PSp, and PPv) and posterior tuberculum (SG, P, PGl, PGm, and TLa) also have higher relative degree in the freezer network than the evading freezers. There are also some notable overlaps in nodes with high degree in the evading freezers and freezers, such as parts of the telencephalon like the central, posterior, and lateral portions of the dorsal telencephalon (Dc, Dp, and Dl, respectively) and posterior and ventral zone of the ventral telencephalon (Vc and Vd-vd, respectively). In general, there is more overlap in the functional brain networks of evading freezers and freezers within the telencephalon than beyond.

## Discussion

The present study is the first to unambiguously identify the presence of individual behavioral variation in the fear reactivity of zebrafish. We built on prior work that found fish exhibit a mix of active and passive responses to CAS. We can now add that individuals express fear memories in different ways: some individuals have high levels of freezing interspersed with normal swimming whereas others intersperse evasive behavior with freezing. Finally, we uncovered patterns of brain activity that differentiate these behavioral types, finding that regions outside the telencephalon, such as the cerebellum, posterior tuberculum, preoptic area, hypothalamus, and pretectum, largely underlie individual differences in fear memory behavior.

We found that fish respond to CAS with an increase in both freezing and evasive behaviors, with freezing being the more robust and persistent response that is less influenced by strain and sex. These responses to CAS itself are consistent with previous reports of active and passive responses in fish (Hall and Suboski, 1995; Maximino et al., 2018a; Speedie and Gerlai, 2008; von Frisch, 1938). We also found that strain had a modest effect on behavior, with the most prominent finding being that TU fish were overrepresented in the non-reactive cluster during fear memory expression. This echoes prior work where the TU strain was found to have a faster rate of contextual fear memory extinction than the AB and TL strains (Kenney et al., 2017). We also saw a sex difference with respect to evasive behaviors, where males appear to increase their evasive behaviors more than females when CAS is present. Freezing, however, is largely similar across sexes. This is consistent with a recent study that found both male and female fish increase freezing in response to CAS (Akinrinade et al., 2023). However, the prior study found no increase in evasive behaviors in response to CAS, and no sex differences. We suspect that this may be because in the present study we assessed evasive behavior as a percentage of time not freezing whereas the prior work examined levels of evasive behavior as a proportion of total time. We took our approach because many fish spend more than half the time freezing in response to CAS, and so the opportunity for other behaviors is limited. By examining evasive behavior as the percentage of time spent not freezing (i.e., active time), we have greater sensitivity to uncover subtle behavioral differences.

A key finding from the present study was the identification of individual differences in how fish behave during fear memory recall. We found that fish behavior falls into one of four groups: non-reactive, evaders, evading freezers, and freezers. The evading freezer and freezer groups are the most clearly associated with fear memory. This is because there were a substantial number of animals that fell into the evader category in the vehicle treated fish and the CAS treated fish during pre-exposure (Figure 5A). This suggests that an increase in evasion in the absence of freezing may be a behavioral response to netting and placement in the tank rather than to the alarm substance (Shishis et al., 2023). We suspect this elevated evasive behavior may be due to the small size of the tank used in this study. We chose a small tank size to limit the amount of CAS needed to elicit a fear response.

Our findings on individual differences in fear memory expression are distinct from prior work examining such differences in rodents and fish that classify animals as being either high or low in fear expression. For example, using an active avoidance paradigm with CAS, Maximino and colleagues (Maximino et al., 2018a) identified zebrafish as high or low in avoidance during memory expression, finding that high avoiders had higher levels of freezing and lower levels of erratic movements during CAS exposure. A similar approach has been taken in rats, where individual differences have been identified by classifying animals as either high or low in fear based on their freezing responses to conditioned stimuli (Graham and Richardson, 2016; Walters et al., 2016). Interestingly, rats have been found to exhibit a mix of active (darting) and passive (freezing) fear behaviors during fear conditioning to an electric shock where females exhibit more darting than males (Gruene et al., 2015; Mitchell et al., 2022). Elevated darting was also associated with less freezing in rats. These findings mirror some of the findings in the present study, such as the presence of both active (evasive) and passive (freezing) fear behaviors. However, we do not find as clear of a sex difference in active and passive behaviors during fear memory expression, perhaps due to the use of a different unconditioned stimulus (CAS versus electric shock) and/or species.

The cerebellum stands out from our findings as being central to freezing behavior. This conclusion is supported by both the PLS and network analysis. PLS analysis found that neural activity of several cerebellar regions (CC, CCe-m, LCa, and MON) strongly contributed to the covariation of brain activity with freezing behavior. The network analysis also identified the CC as a hub with many unique interactions with the pallium and subpallium. Relatedly, the inferior olive (IO) was also identified as important by the PLS and network analysis. The IO is a key source of instructive input to the Purkinje cells of the cerebellum that process information and serve as its primary output (De Zeeuw et al., 1998; Dohaku et al., 2019; Pose-Méndez et al., 2023). A role for the cerebellum in fear-induced freezing is consistent with prior work in goldfish (Yoshida et al., 2004) and recent views of the cerebellum playing an important role in socio-emotional behavior alongside its more well established involvement in motor coordination (Strata, 2015). In looking closer at the functional connectivity of the cerebellum in freezers, we find connections between the CC, IO, and Dm. The Dm is thought to be the teleostan homolog of the basolateral amygdala (Mueller, 2022; Porter and Mueller, 2020) which has been implicated in fear memory learning in both zebrafish and rodents (Fanselow and LeDoux, 1999; Lal et al., 2018; Sun et al., 2020).

Another collection of brain regions that stands out as key to freezing behavior are the posterior tuberculum and pretectum. Within the posterior tuberculum, the preglomerular nuclei are amongst the top contributors to freezing in the PLS and network analysis (particularly the PGm and PGl). These preglomerular nuclei receive a variety of sensory inputs and project to the pallium, making it functionally similar the mammalian dorsal thalamus (Mueller, 2012; Wullimann, 2020). In particular, the PGm in goldfish, a related teleost species, receives chemosensory input (Northcutt, 2006); this may be important for the memory of prior exposure to CAS. Pretectal nuclei, like the PSp and PPv, were also identified as important by both the PLS and network analysis. The pretectum is key to visual processing as these regions receive retinal input and have reciprocal interactions with the optic tectum (Yáñez et al., 2018). Interestingly, several pretectal nuclei, including the PPv, receive or send projections to the CC of the cerebellum (Yáñez et al., 2018). Taken together, these data suggest that the freezing behavior may be driven first by visual information of the conditioning tank which elicits a chemosensory memory of the CAS, and subsequently engages a cerebellar-telencephalic circuit to initiate the coordination of freezing behavior. Further anatomical and functional work would be required to more clearly delineate the functions of these circuits in the context of freezing behavior.

In the functional brain network of evading freezers, we see greater engagement of the hypothalamus and preoptic areas. For example, the network analysis identified the SC, PPp, and PPa, parts of the preoptic area, as high in degree and for having a higher proportion of unique edges than other brain regions. These regions formed a cluster that has many unique edges to hypothalamic and subpallial regions (Figure 8A). The preoptic area of zebrafish has been found to be a neurosecretory area, roughly homologous to the paraventricular nucleus in mammals (Herget et al., 2014). This region, along with other hypothalamic nuclei, modulate a broad spectrum of behaviors and physiological processes, including the hypothalamic-pituitary-interrenal (HPI) axis that regulates the release of cortisol, a central stress hormone (Wendelaar Bonga, 1997). Relatedly, CAS has been found to increase cortisol in zebrafish (Abreu et al., 2017); and in the frillfin goby (*Bathygobius soporator*) cortisol modulates the behavioral effect of CAS (Barreto et al., 2014). Thus, it may be the case that individual variation in the status of the HPI axis in zebrafish may help determine if zebrafish exhibit a combination of evasive and freezing behaviors (i.e., evading freezers) or freezing interspersed with normal swimming (i.e., freezers).

In conclusion, we have identified the presence of individual behavioral variation in how zebrafish express fear memories. We find that the strain and sex of fish affect fear responses and that the behavioral response during learning is predictive of memory expression. Behavioral variation is driven by differences in the activity of brain regions outside the telencephalon, such as the cerebellum, preglomerular nuclei, preoptic area and hypothalamus. Future work will be needed to probe the function of these circuits and determine how they may be differentially wired to give rise to variation in behavior.

## Materials and Methods

### Subjects

Animals were female and male zebrafish from the AB, TU, TL and WIK strains 6 to 9 months of age. Fish were bred in-house and raised at Wayne State University and were within two generations of fish obtained from the Zebrafish International Resource Center (ZIRC - University of Oregon). Fish were housed in a re-circulating system (Aquarius Fish System, Aquatic Enterprises) with a 14/10-hour light/dark cycle (lights on at 8:00 a.m.; pH 7.5 ± 0.2 °C; water conductivity 500 ± 10 μS; temperature 27.5 ± 0.5 C). Fish were fed two times a day with dry feed in the morning (Gemma 300, Skretting) and brine shrimp in the afternoon (Artemia salina; Brine Shrimp Direct. All the procedures were approved by the Wayne State University Institutional Cara and Use Committee (Protocol ID: 21-02-3238).

For the behavioral experiments, animals were housed in male/female pairs (2 female/male pairs per tank separated by a transparent divider) in a 2L tank for one week before behavioral manipulations. The pair-housing system minimizes potential stress induced by social isolation (Parker et al., 2012), and maintains a consistent environment while allowing for the identification of individual fish across days. Behavioral experiments were performed between 11:00 and 15:00 h. Fish were moved to the behavioral room 1 hour before experiments to allow animals to adapt to the new environment. The fear conditioning task consisted of 3 days of behavioral testing: day 1: habituation; day 2: conditioning; day 3: memory recall (Figure 1A). After behavioral recording, animals remained in the room for 1 hour before being moved back to the re-circulating system. Sex was determined using three secondary sex characteristics: body shape, color, and presence of tubercles on the pectoral fins (McMillan et al., 2015). At the end of the experiment, we confirmed sex after euthanization by dissection to identify the presence of eggs.

To ensure data reliability, eight independent batches of offspring for each strain were used for behavioral testing (n = 12 – 16 per strain in each batch). All behavioral testing was carried out using a counterbalanced design across sex and placement in tank (front/back).

### Fear conditioning

The fear conditioning protocol was adapted from prior work (Kenney et al., 2017) to use the Zantiks automated system (Zantiks Ltd) and conspecific alarm substance (CAS) as a fear conditioning unconditioned stimulus (Maximino et al., 2018b). Two tanks (20 × 7 × 9 cm) were recorded simultaneously during the experiments at 60 fps. A pilot study was performed to determine the best CAS concentration starting with previous work in which a concentration of 3.5mL/L elicited fear-related responses up to 7 days after exposure (Maximino et al., 2018b). We found a 1:10 dilution of this dosage (0.35 mL/L) elicited a robust freezing response with some variation on memory day (Figure S1). Fish were tracked using DeepLabCut (Mathis et al., 2018) by labelling three points (head, trunk and tail). We used ResNet101 network and trained a deep neural network on 200 manually labelled frames (divided across the 4 strains and both sexes), the network was then refined and improved by correcting outliers to include 578 additional frames.

### CAS extraction

CAS is a substance released by fish when their epithelial cells are damaged, typically in response to a strike from a predator. We extracted CAS as previously described (Egan et al., 2009; Quadros et al., 2016). Briefly, CAS was obtained from donor fish that were euthanized via rapid cooling in 4 °C water. Fish were placed in a 10 cm petri dish and epidermal cells were damaged via 10 shallow incisions on both sides of the fish body using a surgical blade on ice without drawing blood. 10 mL of distilled water was then added to the dish and it was gently agitated to ensure complete coverage of the lacerated areas on the fish body. Because 4 different strains were used for the behavioral experiments, we prepared stock solutions of alarm substance by mixing extract from 1 fish per sex/strain and then aliquoting before storing at −20 °C for no longer than 4 weeks.

### Random forest for automated behavioral identification

To automate capture of behavior we developed a random forest machine learning model. After fish were tracked using DeepLabCut, we calculated postural information for each individual, extracting 34 parameters (Table S1) over a sliding window of 750 ms (45 frames). We manually labelled 14199 frames per behavior for training for the following behaviors: (1) freezing, the absence of movement with fast opercular movements, (2) erratic movements, zig-zagging movement of at least 3 quick C-bends, (3) burst swimming, a rapid increase of speed in one direction, 4) straight swimming, normal speed swimming in one direction and, (5) normal turns, (Figure 1B). We used an 80/20 (train/test) split for training and testing the model.

To optimize the model, we tested a range of variables per level (1–15) and trees. We found that 10 variables and 2000 trees yielded the lowest out of bag error rate (2.88%). Results of testing on 20% of unseen data also yielded excellent results with errors of no more than 6% and typically much lower (Figure 1C).

### Identification of behavioral clusters

Clusters were identified by creating a k-nearest neighbor network and then applying the Louvain community detection algorithm (Blondel et al., 2008). We only used behavior on memory day from fish exposed to CAS. To calculate distances for the k-nearest neighbor network, we first standardized the behavioral data (% freezing, % evasion, and % evasion as a proportion of active time). We then calculated similarity scores between individual fish:

$$Similarity\;score\;=\frac{1}{1\;+\;D}$$

where D is the Euclidean distance between each fish in three-dimensional behavioral space. We selected the optimal k value by calculating internal clustering metrics at each value of k: Calinski–Harabsz index (Caliński and Harabasz, 1974), silhouette index (Rousseeuw, 1987), and Davies–Bouldin index (Davies and Bouldin, 1979). Based on these data, we chose k=74, which fell within a range that optimized internal clustering metrics and showed robustness to small changes in k (Figure S2). Clustered data were visualized using a uniform manifold approximation (UMAP) (McInnes et al., 2020) as implemented in the UMAP R package (version 0.2.10).

To identify clusters in new data, such as during pre-exposure and exposure or in fish used for brain imaging analysis, we first standardized the new data using the parameters from the initial clustering described above. We then assigned clusters to each fish based on the number of connections to its 33 nearest neighbors in the initial network. Thirty-three was chosen because it is half the size of the smallest cluster identified.

### Tissue clearing and staining

The brains were collected fifteen minutes after fear memory expression. Fish were rapidly euthanized via submersion in ice cold water and heads were removed and fixed in 4% paraformaldehyde (PFA) overnight. Animals that were assigned the wrong sex were removed from data analysis, as well as its paired fish (< 2%). After overnight fixation, brains were carefully dissected in cold PBS. Brains damaged during the dissections were discarded. Tissue staining and clearing was performed using iDISCO+ (Renier et al., 2016) as previously described (Rajput et al., 2025). After dissections, samples were washed three times with PBS (30 min washes) at room temperature followed by dehydration using methanol/water (20, 40, 60, 80 and 100% methanol for 30 min each). Samples were placed in 100% methanol for one hour and chilled in the fridge (4° C). They were then incubated at 4° C overnight in 5% hydrogen peroxide in methanol. The next day, samples were rehydrated in methanol/water (80, 60, 40 and 20%, 30 min each) followed by two PBS washes, one PTx.2 (PBS with 0.2% TritonX-100) of one hour each and an overnight incubation in 5xSSCT buffer (sodium chloride sodium citrate and 0.2% TritonX-100). The following day, an acetylation step was performed with 0.25% v/v acetic anhydride for 30 min followed by three washes with ultrapure water for 5 min each. Samples were then incubated in hybridization buffer (Molecular Instruments) for 15 min at room temperature followed by one hour incubation at 37° C with the same buffer. A solution of hybridization buffer and *cfos* probes (2 pmol per sample) was then prepared and samples were incubated with the probes for 3 days. Samples were then washed three times using probe wash buffer (Molecular Instruments) followed by two 5xSSCT washes of one hour each. Finally, samples were incubated for 1 h with amplification buffer (5×SSC, 0.1% Tween 20, 10% dextran sulfate) and then incubated in the dark for 3 days in 125 μL of amplification buffer and 2.5 μL of each B1 hairpin (7.5 pmol). After amplification, samples were kept in the dark and washed five times in 5xSSCT buffer and left overnight. Clearing then began with dehydration in methanol/water mixtures (20, 40, 60, 80, 100, and 100%) for one hour each, followed by three hours incubation in dichloromethane/methanol (66%/33%) solution. Samples were then incubated two times for 15 min with dichloromethane. Lastly, the solution from the tubes was removed and dibenzyl ether was added to the samples in which they remained until imaging.

### Whole-brain imaging

Cleared samples were imaged via light-sheet microscopy on an UltraMicroscope II (Miltenyi Biotec). Samples were immersed in dibenzyl ether and affixed to a pedestal using a refractive index matched ultraviolet cured resin (NOA 61, Norland Products). Images were acquired at 5× magnification. Illumination of samples was from the right laser using dynamic horizontal focusing and a step size of 4 μm. Autofluorescence images were acquired using a 480 nm laser for registration and *cfos* images were acquired using an illumination laser of 641 nm. The exposure time was 200 ms, sheet width of 50%, and a sheet numerical aperture of 0.156. Images were acquired as 1 × 2 tiles and stitched using TeraStitcher (Bria and Iannello, 2012).

### Automated cell detection

Detection of *cfos* positive cells was done using CellFinder, version 0.4.20 (Tyson et al., 2021) as previously described (Rajput et al., 2025). Briefly, cell candidate detection was first performed and those cell candidates that were closer than 9 μm to one another were removed. We then applied the CellFinder artificial neural net supervised machine learning algorithm to differentiate cells from non-cells. To train the CellFinder network, we labelled 25,460 cells and non-cells and trained the network to an accuracy of 97.7%.

### Registration to AZBA

Images were registered to the adult zebrafish brain atlas (AZBA) using advanced normalization tools (version 2.4.4; Avants et al., 2011) to automatically parcellate brains into individual brain regions as previously described (Rajput et al., 2025). We initially generated a template from autofluorescence images that was a combination of a previously generated template (Rajput et al., 2025) and 10 brains from the present study. The autofluorescence image from AZBA was then registered to the template and the transformation from each individual brain to the template was used to bring the AZBA autofluorescence image into the space of individual brains.

### Partial least squares (PLS) analysis

We implemented PLS analysis (Krishnan et al., 2011) in R to identify covariation between behavior and regional brain activity. Because of batch-to-batch variation in the brightness of *cfos* images, cell counts were normalized based on the median overall *cfos* count for each cohort. This normalized data was then used for PLS (n = 87). Only fish exposed to CAS during training day were included in the analysis. Using mean-centered behavioral PLS correlation, we applied a singular value decomposition to a matrix of correlations between normalized *cfos* counts and four behaviors: % normal swimming, % freezing, % abnormal swimming, and % abnormal swimming while active (i.e., not freezing). To determine which latent variables to consider, we applied permutation tests (resampled 10,000 times without replacement) and used this to calculate p-values associated with each singular value. To determine the reliability of brain region contributions to a specific latent variable, we used bootstrapping (resampled 10,000 times with replacement). Bootstrap ratios were calculated by dividing the salience associated with each brain region by its standard error calculated from bootstrapping making these ratios similar to z-scores.

### Brain network analysis

Brain networks were derived from Pearson correlation matrices capturing covariation between *cfos* counts per brain region across animals in each experimental group. Matrices were thresholded using a false discover rate (FDR) of 0.001. For calculating the initial p-values from Pearson’s correlations, we used the lowest number of observations (n = 16) from the groups used for making networks. We made this conservative correction because the p-value is highly sensitive to the number of observations, which would cause a large skew in the number of significant edges identified for each group. To capture edges that were unique to each network, we identified edges whose 95% confidence intervals did not overlap between networks. Only those edges that were significant (i.e., FDR < 0.001) and did not have overlapping confidence intervals were labelled as ‘unique’.

Clustering of graphs was done by identifying the partition that optimized modularity. Relative degree scores for evading freezer and freezer networks were calculated using the following equation:

$${\mathrm{Relative}\;\mathrm{degree}\;\mathrm{score}}_{i}=\frac{D_{i,Fr}\;-\;D_{i,EF}}{D_{i,Fr}\;+\;D_{i,EF}}$$

Where $D_{i,Fr}$ and $D_{i,EF}$ are the degree of the *i*th node for the freezer and evading freezer groups, respectively. Networks were visualized using Cytoscape version 3.10.2 (Shannon et al., 2003; Su et al., 2014).

### Coding and statistics

Analysis was performed using R version 4.3.3. To identify differences at specific time points during behavior we used independent samples t-tests and compared CAS and vehicle treated groups. One sample t-tests were used to compare difference scores to zero. Permutation resampling without replacement (10,000 times) was used to assess significance of strain and sex for each behavioral cluster. We used the igraph (Csardi and Nepusz, 2006; version 2.02) package for network analysis and the cccd (version 1.6) package to build the k-nearest neighbor networks. The ClusterCrit package (version 1.3.0) was used to assess internal clustering metrics.

## Supplementary Material

Supplement 1 Figure S1. Freezing behavior in response to different concentrations of CAS.
Figure S2. Internal clustering metrics for K-nearest neighbor partitions.
Figure S3. Bootstrap ratios for PLS analysis.
Figure S4. Functional network for non-reactive zebrafish.

Supplement 2 Table S1. Parameters used for training random forest machine learning model.
Table S2. Brain region abbreviations.
