# Supplementary material for: Individual differences in fear memory expression engage distinct functional brain networks
Source: bioRxiv. 2025 Jul 28:2025.05.12.653531. Originally published 2025 May 13. Preprint. [Version 2] doi: 10.1101/2025.05.12.653531 (PMC12132308; doi:10.1101/2025.05.12.653531)
Supplement: Supplement 1 — Figure S1. Freezing behavior in response to different concentrations of CAS. Figure S2. Internal clustering metrics for K-nearest neighbor partitions. Figure S3. Bootstrap ratios for PLS analysis. Figure S4. Functional network for non-reactive zebrafish. [file media-1.pdf]

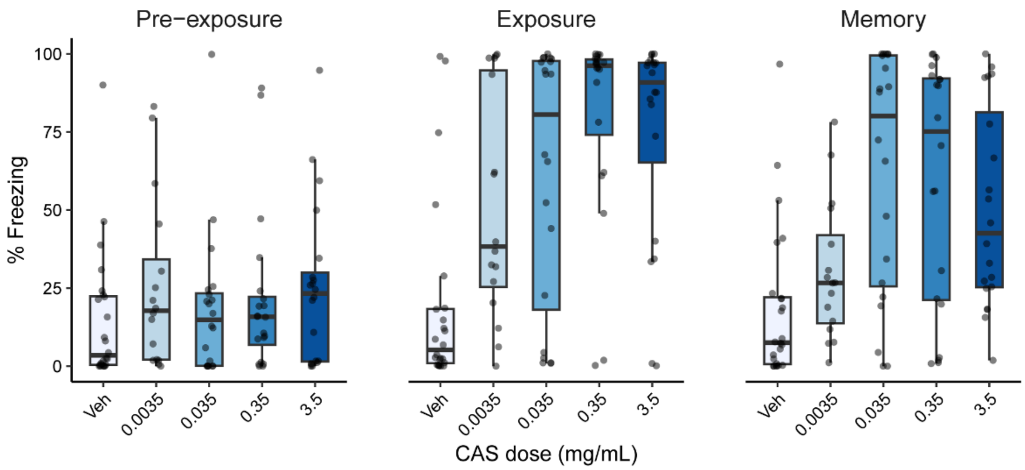

Figure S1. Freezing in response to different doses of CAS during pre-exposure, exposure, and memory day.

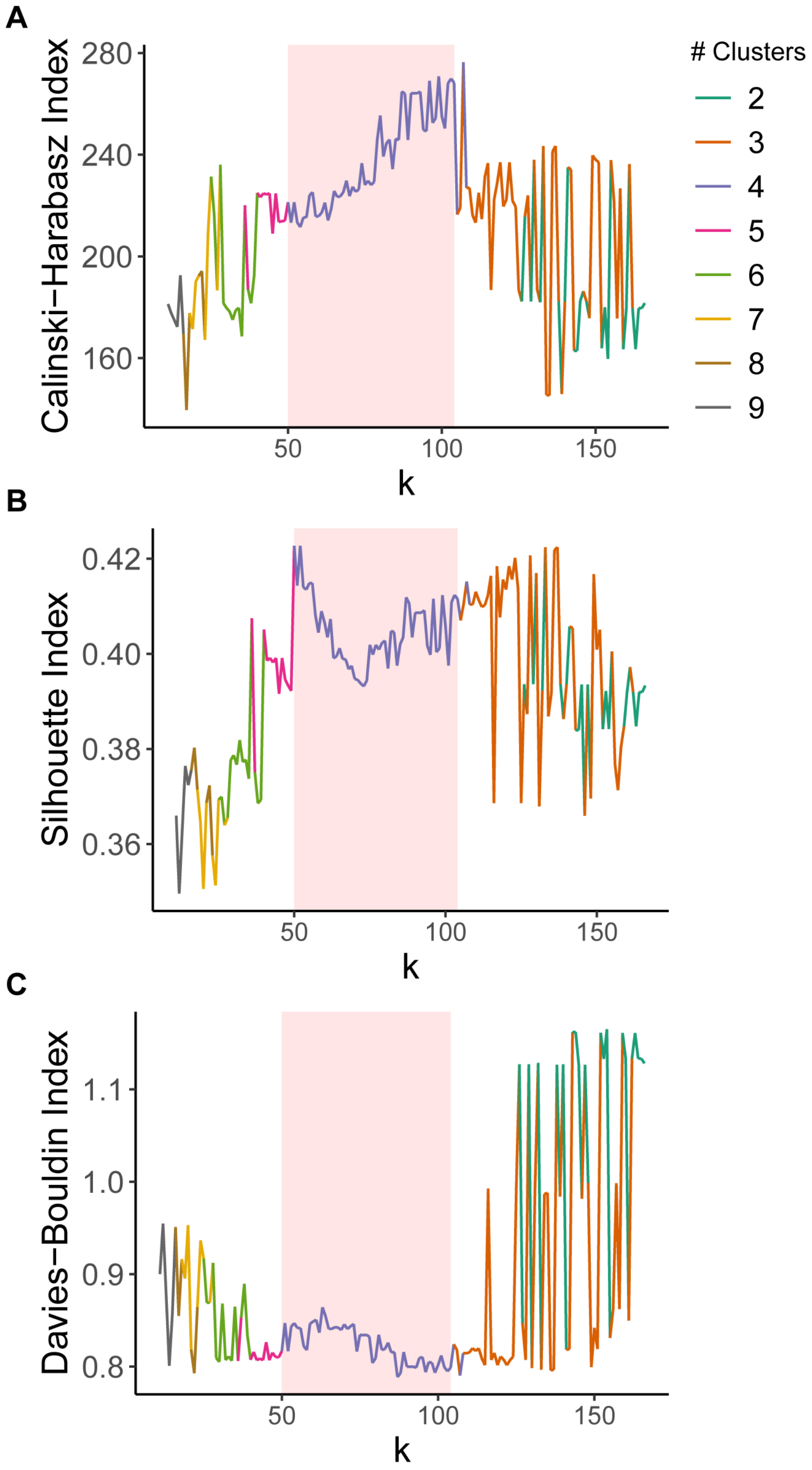

Figure S2. Internal clustering metrics applied to K-nearest-neighbor network partitions at different values of k. The metrics are A) the Calinski-Harabasz, B) Silhouette, and C) Davies-Bouldin indices. Red shaded area marks a region high in clustering and stability.

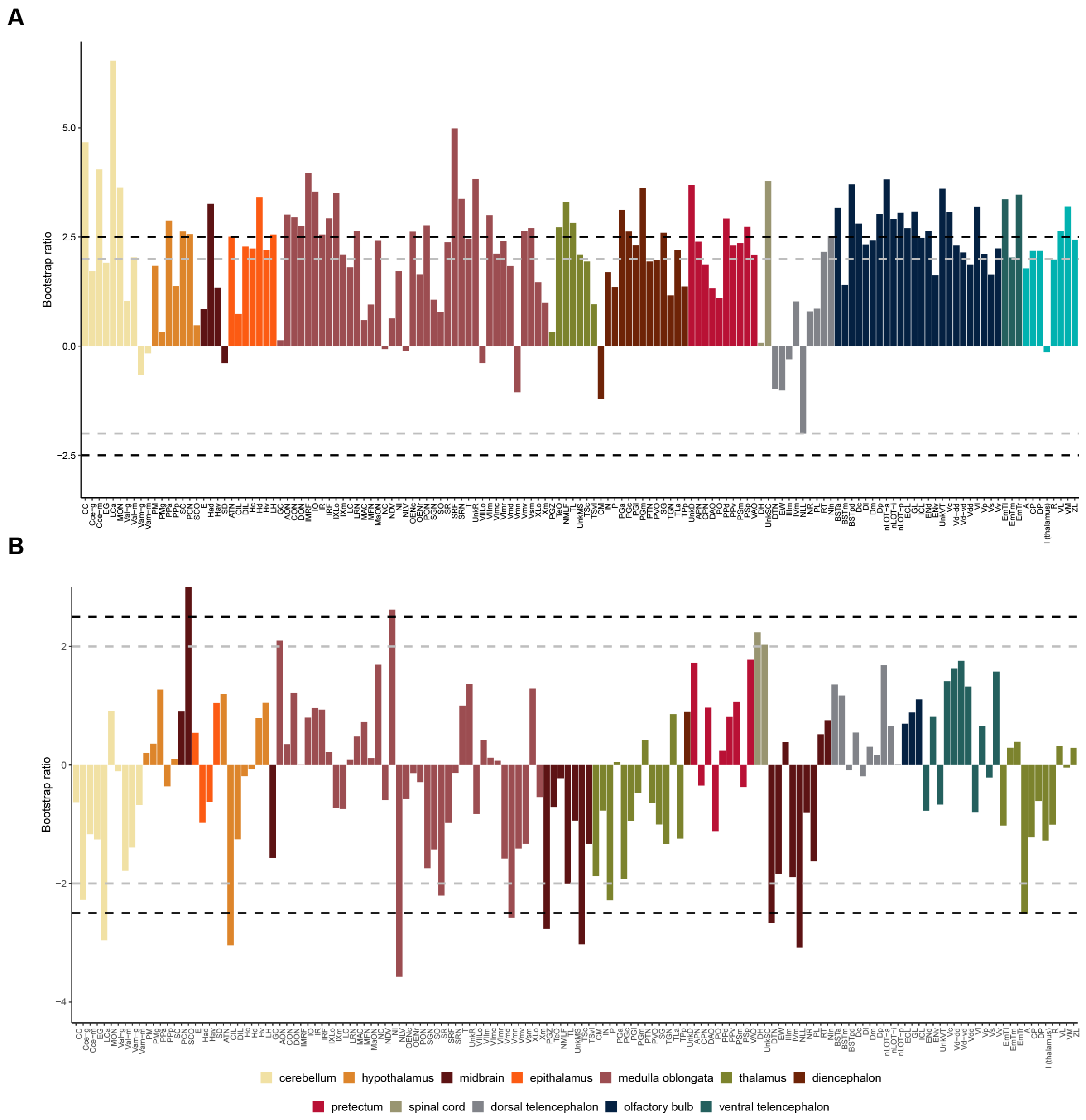

Figure S3. Bootstrap ratios from PLS analysis for the first (A) and second (B) contrasts.

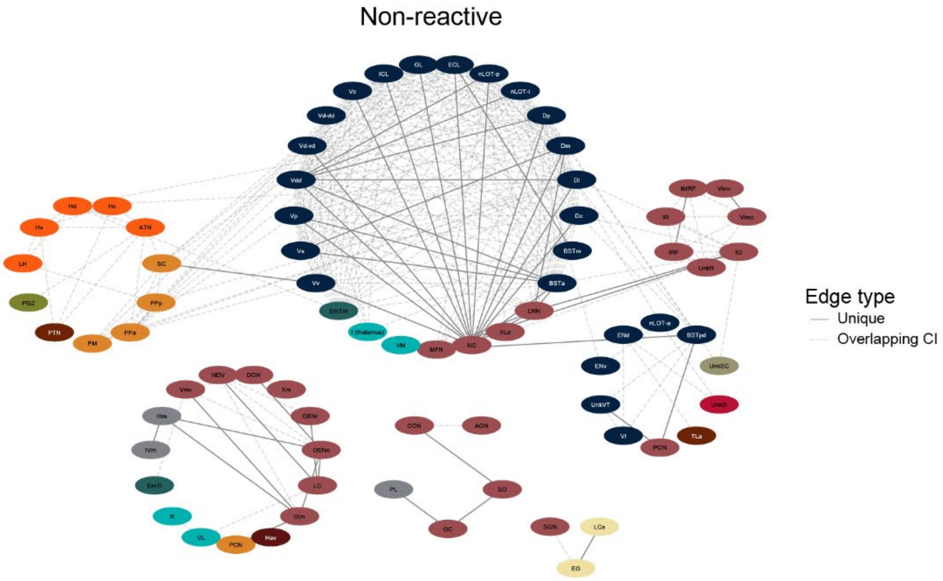

Figure S4. Functional network for non-reactive zebrafish. Nodes represent brain regions and edges represent suprathreshold correlations (FDR < 0.001). Nodes follow the same coloring scheme as in Figure 7.
