# Supplementary material for: Individual differences in fear memory expression engage distinct functional brain networks
Source: bioRxiv. 2025 Jul 28:2025.05.12.653531. Originally published 2025 May 13. Preprint. [Version 2] doi: 10.1101/2025.05.12.653531 (PMC12132308; doi:10.1101/2025.05.12.653531)
Supplement: Supplement 2 — Table S1. Parameters used for training random forest machine learning model. Table S2. Brain region abbreviations. [file media-2.pdf]

Table S1. Parameters used for training the random forest model from three points (head, trunk, and tail) tracked using DeepLabCut.

Parameter number	Parameter
1	Number of turns
2	Absolute turning angle
3	Net turning angle
4	Absolute angular velocity
5	Net angular velocity
6	Absolute angular acceleration
7	Net angular acceleration
8	Distance between head and tail
9	Distance between head and trunk
10	Distance between trunk and tail
11	Absolute tail velocity, x
12	Net tail velocity, x
13	Absolute tail velocity, y
14	Net tail velocity, y
15	Absolute trunk velocity, x
16	Net trunk velocity, x
17	Absolute trunk velocity, y
18	Net trunk velocity, y
19	Absolute head velocity, x
20	Net head velocity, x
21	Absolute head velocity, y
22	Net head velocity, y
23	Absolute tail acceleration, x
24	Net tail acceleration, x
25	Absolute tail acceleration, y
26	Net tail acceleration, y
27	Absolute trunk acceleration, x
28	Net trunk acceleration, x
29	Absolute trunk acceleration, y
30	Net trunk acceleration, y
31	Absolute head acceleration, x
32	Net head acceleration, x
33	Absolute head acceleration, y
34	Net head acceleration, y

Table S2. Gray matter regional abbreviations, names, and ontological levels.

Abbreviation	Name	Ontological level
A	anterior thalamic nucleus	thalamus
AON	anterior octaval nucleus	medulla oblongata
APN	accessory pretectal nucleus	pretectum
ATN	anterior tuberal nucleus	hypothalamus
BSTa	bed nucleus of the stria terminalis, anterior division	telencephalon
BSTm	bed nucleus of the stria terminalis, medial division	telencephalon
BSTpd	bed nucleus of the stria terminalis, posterior division	telencephalon
CC	cerebellar crest	cerebellum
Cce-g	cerebellar corpus, granular layer	cerebellum
Cce-m	cerebellar corpus, molecular layer	cerebellum
CIL	central nucleus of the inferior lobe	hypothalamus
CM	mammillary body	posterior tuberculum
CON	caudal octavolateralis nucleus	medulla oblongata
CP	central posterior thalamic nucleus	thalamus
CPN	central pretectal nucleus	pretectum
DAO	dorsal accessory optic nucleus	pretectum
Dc	central zone of dorsal telencephalon area	telencephalon
DH	dorsal horn	spinal cord
DIL	diffuse nucleus of the inferior lobe	hypothalamus
Dl	lateral zone of the dorsal telencephalon	telencephalon
Dm	medial zone of dorsal telencephalon	telencephalon
DON	descending octaval nucleus	medulla oblongata
Dp	posterior zone of dorsal telencephalon area	telencephalon
DP	dorsal posterior thalamic nucleus	thalamus
DTN	dorsal tegmental nucleus	tegmentum
E	epiphysis	epithalamus
ECL	external cellular layer of olfactory bulb	telencephalon
EG	granular eminence	cerebellum
EmTl	lateral thalamic eminence	thalamic eminence
EmTm	medial thalamic eminence	thalamic eminence
EmTr	rostral thalamic eminence	thalamic eminence
ENd	entopeduncular nucleus, dorsal part	telencephalon
ENv	entopeduncular nucleus, ventral part	telencephalon
EW	Edinger-Westphal nucleus	tegmentum
GC	central gray	medulla oblongata
GL	glomerular layer of olfactory bulb	telencephalon
Had	dorsal habenular nucleus	epithalamus
Hav	ventral habenular nucleus	epithalamus

Hc	caudal zone of periventricular hypothalamus	hypothalamus
Hd	dorsal zone of periventricular hypothalamus	hypothalamus
Hv	ventral zone of periventricular hypothalamus	hypothalamus
I (thalamus)	Intermediate thalamic nucleus	thalamus
IAF	inner arcuate fibers	
ICL	internal cellular layer of olfactory bulb	telencephalon
IMRF	intermediate reticular formation	medulla oblongata
IN	Intermediate nucleus	posterior tuberculum
IO	inferior olive	medulla oblongata
IR	inferior raphe	medulla oblongata
IRF	inferior reticular formation	medulla oblongata
LC	locus coeruleus	medulla oblongata
LCa	caudal lobe of cerebellum	cerebellum
LH	lateral hypothalamic nucleus	hypothalamus
LRN	lateral reticular nucleus	medulla oblongata
MAC	Mauthner cell	medulla oblongata
MaON	magnocellular octaval nucleus	medulla oblongata
MFN	medial funicular nucleus	medulla oblongata
MON	medial octavolateralis nucleus	cerebellum
NC	commissural nucleus of Cajal	medulla oblongata
NDV	nucleus of the descending trigeminal root	medulla oblongata
NI	isthmic nucleus	medulla oblongata
NIn	interpeduncular nucleus	tegmentum
NLL	nucleus of the lateral lemniscus	tegmentum
nLOT-a	nucleus of the lateral olfactory tract, anterior part	telencephalon
nLOT-i	nucleus of the lateral olfactory tract, intermediate part	telencephalon
nLOT-p	nucleus of the lateral olfactory tract, posterior part	telencephalon
NLV	nucleus lateralis valvulae	medulla oblongata
NMLF	nucleus of the medial longitudinal fascicle	midbrain
NR	red nucleus	tegmentum
OENc	octavolateralis efferent neurons, caudal part	medulla oblongata
OENr	octavolateralis efferent neurons, rostral part	medulla oblongata
P	posterior thalamic nucleus	posterior tuberculum
PCN	paracommissural nucleus	diencephalon
PGa	anterior preglomerular nucleus	posterior tuberculum
PGc	caudal preglomerular nucleus	posterior tuberculum
PGl	lateral preglomerular nucleus	posterior tuberculum
PGm	medial preglomerular nucleus	posterior tuberculum
PGZ	periventricular gray zone of optic tectum	midbrain
PL	perilemniscal nucleus	tegmentum
PM	magnocellular preoptic nucleus	diencephalon

PMg	gigantocellular part of magnocellular preoptic nucleus	diencephalon
PO	posterior pretectal nucleus	pretectum
PON	posterior octaval nucleus	medulla oblongata
PPa	parvocellular preoptic nucleus, anterior part	diencephalon
PPd	periventricular pretectal nucleus, dorsal part	pretectum
PPp	parvocellular preoptic nucleus, posterior part	diencephalon
PPv	periventricular pretectal nucleus, ventral part	pretectum
PSm	magnocellular superficial pretectal nucleus	pretectum
PSp	parvocellular superficial pretectal nucleus	pretectum
PTN	posterior tuberal nucleus	posterior tuberculum
PVO	paraventricular organ	posterior tuberculum
R	rostrolateral nucleus	thalamus
RT	rostral tegmental nucleus	tegmentum
SC	suprachiasmatic nucleus	diencephalon
SCO	subcommissural organ	diencephalon
SD	dorsal sac	epithalamus
SG	subglomerular nucleus	posterior tuberculum
SGN	secondary gustatory nucleus	medulla oblongata
SO	secondary octaval population	medulla oblongata
SR	superior raphe	medulla oblongata
SRF	superior reticular formation	medulla oblongata
SRN	superior reticular nucleus	medulla oblongata
T	tangential nucleus	medulla oblongata
TeO	optic tectum	midbrain
TGN	tertiary gustatory nucleus	posterior tuberculum
TL	longitudinal torus	midbrain
TLa	lateral torus	posterior tuberculum
TPp	periventricular nucleus of posterior tuberculum	posterior tuberculum
TSc	central nucleus of semicircular torus	midbrain
TSvl	ventrolateral nucleus of semicircular torus	midbrain
Val-g	lateral division of valvula cerebelli, granular layer	cerebellum
Val-m	lateral division of valvula cerebelli, molecular layer	cerebellum
Vam-g	medial division of valvula cerebelli, granular layer	cerebellum
Vam-m	medial division of valvula cerebelli, molecular layer	cerebellum
VAO	ventral accessory optic nucleus	pretectum
Vc	central nucleus of ventral telencephalon area	telencephalon
Vd-dd	dorsal zone of ventral telencephalon	telencephalon
Vd-vd	ventral zone of ventral telencephalon	telencephalon
Vdd	dorsal most zone of ventral telencephalon	telencephalon
Vl	lateral nucleus of ventral telencephalon area	telencephalon
VL	ventrolateral thalamic nucleus	thalamus

VM	ventromedial thalamic nucleus	thalamus
Vp	postcommissural nucleus of ventral telencephalon area	telencephalon
Vs	supracommissural nucleus of ventral telencephalon area	telencephalon
Vv	ventral nucleus of ventral telencephalon area	telencephalon
ZL	zona limitans	thalamus
III	oculomotor nerve	
IIIm	oculomotor nucleus	tegmentum
IVm	trochlear nucleus	tegmentum
Vmd	trigeminal motor nucleus, dorsal part	medulla oblongata
Vmn	mesencephalic nucleus of the trigeminal nerve	medulla oblongata
Vmv	trigeminal motor nucleus, ventral part	medulla oblongata
Vsm	primary sensory trigeminal nucleus	medulla oblongata
VImc	caudal abducens nerve motor nucleus	medulla oblongata
VImr	rostral abducens nerve motor nucleus	medulla oblongata
VIILo	facial lobe	medulla oblongata
VIIm	facial motor nucleus	medulla oblongata
IXLo	glossopharyngeal lobe	medulla oblongata
IXm	glossopharyngeal nerve motor nucleus	medulla oblongata
XLo	vagal lobe	medulla oblongata
Xm	vagal motor nucleus	medulla oblongata
UnkD	unknown diencephalon	pretectum
UnkMS	unknown mesencephalon	midbrain
UnkR	unknown rhombencephalon	medulla oblongata
UnkSC	unknown spinal cord	spinal cord
UnkVT	unknown ventral telencephalon	telencephalon
